# Rates and Determinants of Uptake and Use of an Internet Physical Activity and Weight Management Program in Office and Manufacturing Work Sites in England: Cohort Study

**DOI:** 10.2196/jmir.1108

**Published:** 2008-12-31

**Authors:** Lisa J Ware, Robert Hurling, Ogi Bataveljic, Bruce W Fairley, Tina L Hurst, Peter Murray, Kirsten L Rennie, Chris E Tomkins, Anne Finn, Mark R Cobain, Dympna A Pearson, John P Foreyt

**Affiliations:** ^6^Department of Psychiatry and Behavioral SciencesBaylor College of MedicineHoustonTXUSA; ^5^QuornLeicesterUK; ^4^Unilever Occupational HealthLondonUK; ^3^Tessella Support Services PLCAbingdonOxfordshireUK; ^2^Unilever ResearchColworthBedfordUK; ^1^MiLife Coaching LimitedColworthBedfordUK

**Keywords:** Employee health, Internet, device, behavior change, body weight, psychology, physical activity, occupational health, diet, technology

## Abstract

**Background:**

Internet-based physical activity (PA) and weight management programs have the potential to improve employees’ health in large occupational health settings. To be successful, the program must engage a wide range of employees, especially those at risk of weight gain or ill health.

**Objective:**

The aim of the study was to assess the use and nonuse (user attrition) of a Web-based and monitoring device–based PA and weight management program in a range of employees and to determine if engagement with the program was related to the employees’ baseline characteristics or measured outcomes.

**Methods:**

Longitudinal observational study of a cohort of employees having access to the MiLife Web-based automated behavior change system. Employees were recruited from manufacturing and office sites in the North West and the South of England. Baseline health data were collected, and participants were given devices to monitor their weight and PA via data upload to the website. Website use, PA, and weight data were collected throughout the 12-week program.

**Results:**

Overall, 12% of employees at the four sites (265/2302) agreed to participate in the program, with 130 men (49%) and 135 women (51%), and of these, 233 went on to start the program. During the program, the dropout rate was 5% (11/233). Of the remaining 222 Web program users, 173 (78%) were using the program at the end of the 12 weeks, with 69% (153/222) continuing after this period. Engagement with the program varied by site but was not significantly different between the office and factory sites. During the first 2 weeks, participants used the website, on average, 6 times per week, suggesting an initial learning period after which the frequency of website log-in was typically 2 visits per week and 7 minutes per visit. Employees who uploaded weight data had a significant reduction in weight (−2.6 kg, SD 3.2, *P*< .001). The reduction in weight was largest for employees using the program’s weight loss mode (−3.4 kg, SD 3.5). Mean PA level recorded throughout the program was 173 minutes (SE 12.8) of moderate/high intensity PA per week. Website interaction time was higher and attrition rates were lower (OR 1.38, *P*= .03) in those individuals with the greatest weight loss.

**Conclusions:**

This Web-based PA and weight management program showed high levels of engagement across a wide range of employees, including overweight or obese workers, shift workers, and those who do not work with computers. Weight loss was observed at both office and manufacturing sites. The use of monitoring devices to capture and send data to the automated Web-based coaching program may have influenced the high levels of engagement observed in this study. When combined with objective monitoring devices for PA and weight, both use of the website and outcomes can be tracked, allowing the online coaching program to become more personalized to the individual.

## Introduction

Overweight and obesity are now major causes of preventable health problems across the world. Obesity has serious implications for the individual’s health as well as the population health and economy. A report by the UK National Audit Office (NAO) estimated that, in 1998 alone, the indirect costs of obesity to the UK economy (18 million sick days and 40,000 lost years of working life) were around four times greater (£2.1 billion/year) than the direct costs of treatment (£0.5 billion/year) [1]. A subsequent update by the Health Select Committee in 2004 [2] estimated the cost of obesity to be 27% to 42% higher than the NAO 1998 estimate. These estimates suggest that finding effective interventions that target weight management and physical inactivity within the workplace could potentially be of great value both to employers and to the economy. However, these interventions must be attractive to the employee, scalable, and, most importantly, capable of both initiating and supporting the required behavior change. Interventions must also appeal to employees who are high risk and difficult to reach by other health initiatives, rather than to employees who are already fit and active with a healthy weight.

Health behaviors are personal and complex, and the challenge lies in creating and deploying intervention programs that address this complexity in an engaging, easy to use, and yet effective way. Internet-based interventions serve as a feasible and acceptable delivery method for these programs, thereby providing scale, but evidence suggests that programs must go beyond providing advice and information alone. A review of Internet use for weight loss suggests that successful online programs include a structured approach to modifying energy balance, the use of cognitive-behavioral strategies such as self-monitoring, and individualized feedback and support [3].

Our previous research [4] also shows the importance of interactive design in Internet-based physical activity (PA) motivation programs. Where interactive design is employed, better user engagement and retention are observed. Specifically, in this research [4], the interactive program created higher expectations for PA and increased self-perception of fitness, with increased PA reported in the test group up to 7 months after exposure to the website. In order to test this interactive website with an objective measure of PA, a study was conducted combining the Internet program with a wrist-worn accelerometer [5]. This combination of interactive Web program and monitoring device produced an average increase of 2 hours and 18 minutes of moderate PA per week, with a greater loss of body fat when compared to the control group (wearing the accelerometer but without access to the Web program). To our knowledge, this was the first reported fully automated Internet system with personalized accelerometer feedback to demonstrate increased PA where PA was objectively measured. This study also indicated that engagement with the online program may be important in increasing PA over and above the effects observed from wearing an activity monitor alone.

Following this work with objective PA data collection, we extended the program to include an online weight management module and automated data capture weighing scales. The objective of this study was to assess the level of program engagement of a wide range of employees. A key difference from our previous studies [4,5] was that participants received no payment for taking part in this study. Also, rather than a randomized controlled trial (RCT), the study was conducted in a more naturalistic setting, whereby a branded commercial program (MiLife) was offered to employees as a benefit through collaboration with their company’s occupational health professionals.

## Methods

### Study Design

The study was designed to test the level of engagement with the Web program for employees recruited at 4 work sites in the United Kingdom over a 12-week period and the effects of this on the employees’ health. Work sites were chosen that were geographically and demographically different in order to evaluate if engagement with the program varied by location, baseline demographics, or level of interaction with computers during work hours. Two work sites were in the North West of England and 2 work sites were in the South of England in order to determine the influence of region upon uptake and use of the program. This could be important as, on average, 39% of UK households do not have Internet access. This varies by region, with the highest levels of Internet access observed in the South West and around London, compared to lower levels of access in households in the North West of the country [6]. Additionally, the use of 2 manufacturing sites (1 in the north and 1 in the south) and 2 office-based sites (again in the north and south) allowed the comparison of baseline data from these 4 sites, including an assessment of participant characteristics since research suggests that women may be more likely to participate in work-site health promotion programs than men [7] and that shift work may be a barrier to participation in such programs [8]. The study objective was to assess the use of the Web-based and monitoring device–based PA and weight management program in this range of employees and to determine if engagement with the program was related to the employees’ baseline characteristics or the measured outcomes. This was undertaken with a view to share our experiences when implementing an Internet-based health program in a range of occupational settings, and to build the literature on factors to achieve greater accessibility to and engagement with health support tools.

### Participants

In order to determine the baseline health profiles and characteristics of all employees who were attracted to the MiLife program, minimal exclusion criteria were applied. The program was offered to adults employed at any of the 4 work sites who had regular Internet and email access or who were willing to access Internet and email via computers installed in communal areas at their workplace. Exclusion criteria were pregnancy, any holiday of more than 2 weeks during the study period without computer access, previous instruction from a health professional not to engage in PA, being severely underweight (body mass index, BMI, < 16 kg/m^2^), or already taking part in a clinical trial.

Employees were recruited via leaflet distribution during working hours, including during shift patterns (weekends, nights, etc) at the manufacturing sites. At screening, date of birth, gender, blood pressure (BP), resting heart rate, BMI, and medication were recorded and the Physical Activity Readiness Questionnaire(PAR-Q) [9] and Rose Angina [10] questionnaires were administered. Participants with hypertension (BP ≥ 140/90 mmHg), chronic respiratory conditions, or positive scores on the PAR-Q or Rose Angina questionnaire were not excluded but were required to seek approval from their physician prior to entry into the study.

### Intervention

The Web program was combined with a Bluetooth-enabled wrist-worn triaxial accelerometer to measure PA and with Bluetooth-enabled weighing scales to record body weight (see Multimedia Appendix). Both devices sent the captured data back to the user’s Web program. The data from these devices were reported back to the individual via their automated coaching Web program, allowing self-monitoring of their PA and body weight and also objective measurement of PA and weight throughout the program. The components of the Web program and the monitoring devices are described in more detail below.

Participants recruited to the program were shown, in a brief training session (approximately 20 minutes, group size of up to 15 participants per trainer), how to register on the website and how to create their own personal password-protected account. The trainer also demonstrated how to upload weight data from the scales to the PA monitor and how then to upload the weight and PA data from the PA monitor to the computer. Basic computer skills training was provided when necessary and included use of a mouse, computer startup, log-in, and website navigation.

#### MiLife Web-Based Automated Behavior Change System

The Internet, email, and mobile phone behavior change system was similar to that used in previous studies [4,5]. An introductory series of screens helped participants identify their goals and targets and recommended a suitable program mode (ie, weight loss, weight maintenance, or PA only). A weekly series of screens provided constructive feedback on performance relative to their own target. The system included a weekly schedule (or diary) for planning PA sessions over the next 7 days, for which participants could choose to receive email and/or mobile phone reminders, an approach that has been effective in combination with implementation intentions [11].

The system made recommendations to the user of the mode to follow (weight loss, weight maintenance, or PA) based on their baseline weight, height, waist circumference, and stated goals. The major difference between the modes lies in the frequency with which users are encouraged to monitor their energy intake. For weight loss, monitoring energy intake is frequently encouraged; for weight maintenance, monitoring energy intake is encouraged if weight increases; for PA only, monitoring energy intake plays less of a role. The user could follow the recommended mode, choose another mode, or enter a nonactive browse mode with no goal or target setting. Users could also switch between the modes during the 12-week study period. The tools that support each mode are based on best evidence and practice from the literature. For example, in the weight loss mode, the tools were developed based on strategies used within the Diabetes Prevention Program [12] to promote weight loss and PA, such as self-monitoring, planning, goal setting, and structured feedback.

The design of the Web-based system was founded on key behavior change theories. For example, providing users with information on the typical PA levels of people like themselves is based on Festinger’s (1954) Social Comparison Theory [13], which asserts that individuals engage in social comparison (comparing themselves with others) to evaluate their opinions and abilities. Social Comparison Theory has previously been applied to prevention and health care [14]. Another part of the system offers users solutions for barriers they perceive to be preventing them from taking up healthier behaviors. This is based on Decisional Balance Theory [15], which suggests that if the perceived number of “pros” for a behavior (eg, regular PA) outweigh the “cons” for an individual, then he or she is more likely to perform the behavior.

Other studies have shown that asking people to form specific plans (implementation intentions) [16] about when and how to eat healthily or to be active can increase their levels of healthy eating and PA [17]. The program encourages users to develop implementation intentions via on-screen, diary-style planning tools in which intentions are specified in terms of their date, time, and place, with environmental cueing via the mobile phone SMS text or email reminder service [11]. Community message boards and discussion forums were designed to provide social support, identified as important for behavior change [18].

#### Monitoring Devices: Bluetooth-Enabled PA Monitor and Weighing Scales

 While pedometers are low cost, they are typically used to record walking and therefore are less appropriate for 24-hour monitoring of PA. Accelerometer-based devices tend to allow a wider range of movement and for total PA to be recorded [19-21] in adults [22] and children [23], and they can be worn in several locations on the body, allowing for continuous use.

We developed a water-resistant, Bluetooth, wrist-worn device that could be worn continuously, including while swimming/bathing. The device contained a miniature triaxial accelerometer unit that produced a signal as the wearer made physical movement, recording all movement up to acceleration levels of 6 g. The acceleration signal was measured and the resultant amplitude integrated. The data were then stored within the PA monitor memory ready for download and analysis. To establish validity of the PA monitor, a lab-based study was conducted in 22 adults (12 males, 10 females). Subjects undertook 10 different semistructured PA conditions: lying down, seated computer work, stacking shelves, washing dishes, sweeping, cleaning windows, and 4 treadmill-based activities (walking at 4 km/h and 6 km/h, running at 8 km/h and 10 km/h). These activities were chosen to represent a range of different physical intensities and metabolic equivalents (METs) and included many tasks that may be undertaken by the participants in their daily living. Oxygen uptake (VO_2_)and indirect calorimetry were measured continuously throughout the activities using a K4b2 portable metabolic gas analyzer with data telemetry (Cosmed, Italy) to determine metabolic rate at rest and METs [24] during PA. The activity monitor showed a strong positive correlation with relative VO_2_(left wrist: r = 0.934, *P*< .001; right wrist: r = 0.900, *P*< .001). Receiver operator curves for discrimination of intensity categories showed the activity monitor was able to predict light (MET 1.5-2.99), moderate (MET 3-5.99), and vigorous (MET ≥6) intensity activity when worn on either the left wrist (area under the curve [AUC], sensitivity, specificity: 0.89 [*P*< .001], 91%, 75% for light; 0.86 [*P*< .001], 88%, 81% for moderate; 0.99 [*P*< .001], 91%, 100% for vigorous) or right wrist (AUC, sensitivity, specificity: 0.90 [*P*< .001], 91%, 75% for light; 0.76[*P*= .004], 78%, 71% for moderate; 0.96 [*P*< .001], 95%, 94% for vigorous). Using this data, cut-points were developed to distinguish moderate intensity and vigorous intensity PA. Further validation of these cut-points in a larger sample is planned with the inclusion of non-lab-based activities such as running and walking outside.

Accelerometer data were analyzed by calculating the number of minutes spent within the range corresponding to moderate intensity (MET level 3) or above [24]. Data points were only counted if they were part of a continuous bout of PA of at least 10 minutes within the MET 3+ range. This was following the American College of Sports Medicine guidelines for moderate-intensity aerobic PA, which state that PA can be accumulated toward the 30-minute minimum on 5 days each week by performing bouts each lasting 10 or more minutes [25,26]. In order to represent the underlying signal, the data were smoothed using a moving average filter of width ± 1 point. Modifying the width of the filter had little effect on the results of the analysis.

Data from the PA monitor were transmitted via a Bluetooth microprocessor to a personal computer (PC). Bluetooth-enabled personal weighing scales also sent data on the user’s weight to the PA monitor via Bluetooth. Each weight reading was held within the PA monitor with the PA data until transfer to the PC. The PA monitor memory could store many PA and weight readings before needing to upload the data to the PC. All data were sent via the PC to a central secure database managed by an industry standard commercial infrastructure supplier and held in accordance with the UK Data Protection Act and all relevant regulations. The integration of the PA monitor, weighing scales, and PC permitted direct acquisition of data from the wearer via the Internet throughout the study.

### Outcome Measures and Statistical Considerations

The primary outcome of the study was the assessment of use of the Web-based and monitoring device–based PA and weight management program in this range of employees and the relationship between program use and the secondary outcome measures. Use of the program is defined by nonusage attrition data (Is the subject continuing to use the program?), as discussed by Eysenbach [27], and by the level of engagement (How much is the subject using the program?) assessed through website log-in frequency and log-in duration. The duration of time spent on the website at each log-in was recorded from the time of log-in to the last “click” interaction on the website and did not include dormant time between this last click and the automatic log-out function.

Sample size was more than 200 employees in order to improve the accuracy of the confidence intervals around the expected level of engagement (eg, if 80% are engaged in a study size of 200, the confidence interval is 74-85, while reducing the study size to 100 would widen this confidence interval to 71-87). Allocation of each subject to a program mode (weight loss, weight maintenance, PA, or browse) for data analysis was completed by assessing the number of weeks spent in each mode then allocating the participant to the mode in which he or she had spent the greatest number of weeks. Statistical comparison between the program modes was not undertaken as participants were not randomized to modes and could switch between these modes at any time during the study period.

Secondary outcome measures included baseline characteristics (BMI, health profile, age, gender), weight (data captured from Bluetooth weighing scales), PA level (time spent in moderate and vigorous activity measured via the triaxial accelerometer throughout the 12 weeks), BP, resting heart rate, and sleep quality and quantity (Pittsburgh Sleep Quality Index, PSQI [28]). Weight change for each participant was calculated using the first recorded and the last recorded weight during the 12 weeks, with the last observation carried forward (LOCF). BP and resting heart rate were measured using a Bionet BM5 Vital Signs Monitor (BioNet Laboratories Asia Pte Ltd, Singapore).

The health profile data collected at screening (height, weight, BMI, gender, age, BP) were aggregated for each site and compared to health risk appraisal (HRA) data collected around 3 months earlier at the same sites. As the HRA had included a greater number of employees (n = 992), this was undertaken to test if the employees participating in the Web program were similar to the larger group of employees on the same site who attended the HRA.

At the end of the study, process evaluation questionnaires were sent to managers and occupational health (OH) staff at each site to determine the impact of the study at that site, and feedback questionnaires were sent to all participants. All employees were permitted to keep the hardware and software and, if they wished, could continue to use the program.

Analysis of variance (ANOVA) was used for comparison of baseline data (age, gender, systolic BP, diastolic BP, BMI, resting heart rate) between the sites. Fisher exact test was used to compare the nonusage attrition rates between the sites. The probability of attrition in the first 12 weeks was modeled using multivariate logistic regression with the baseline independent variables (age, gender, baseline systolic BP, baseline diastolic BP, baseline BMI, baseline resting heart rate) and the dependent outcome variables (weight change using LOCF, mean daily recorded minutes of MET 3+ PA, BP change at 12 weeks) with site as a covariate. The association between the total interaction time with the website during the 12 weeks and the baseline independent variables and dependent outcome variables as listed above was modeled using multivariate linear regression, again with site as a covariate. The log of the total interaction time was used to preserve normality assumptions of the model. HRA data were compared with the baseline characteristics of MiLife participants using the Kolmogorov-Smirnov test. Weight change (using LOCF) and BP change at the end of the 12-week study were analyzed using analysis of covariance (ANCOVA), with baseline included as a covariate. Multivariate logistic regression with site, age, and gender as covariates was used to examine the relationship between baseline BP and the probability of attendance at the 12-week BP measure. Data were analyzed using SAS version 9.1 (SAS Institute Inc, Cary, NC, USA).

### Local Research Ethical Review Requirement

The study was approved by 2 independent research ethics committees, one in the North West and one in the South of England. All research was conducted in accordance with the Helsinki Declaration [29].

## Results

### Employee Baseline Characteristics

Of the 2302 employees at the work sites, 265 (12%) agreed to take part in the program. The numbers for each site and the characteristics of these participants are shown in Table 1. The mean age across the sites was 40.9 years (SD 8.1), and the mean BMI at the start of the program was 27.1 kg/m^2^ (SD 4.8). Ethnicity was also recorded to determine appropriate risk of metabolic disease for a given waist circumference and BMI [30]. Of the 265 employees, 13 (5%) were Asian, 3 (1%) were Black, 244 (92%) were White, and the remaining 5 (2%) classified themselves as other or mixed.

**Table 1:** Baseline demographic data for participants at each site

	Office North	Factory North	Office South	Factory South	All Sites
Total number of eligible employees at work site, no.	852	252	705	493	2302
Volunteered for trial, no.	71	44	93	57	265
Percentage of employees that volunteered for trial, %	8	17	13	12	12
Referred to physician, %	32	47	40	49	41
Age (years), mean (SD)	39.5 (7.2)	43.4 (8.2)	39.1^a^ (7.6)	43.7^b^ (8.8)	40.9 (8.1)
Men, %	42	65	31	75	49
BMI (kg/m^2^), mean (SD)	25.7^c^ (4.0)	29.7 (5.6)	26.4^a^ (4.4)	28.4 (4.7)	27.1 (4.8)
Systolic blood pressure (mmHg), mean (SD)	124 (14)	135^d^ (13)	130 (16)	135^a^ (16)	130 (16)
Diastolic blood pressure (mmHg), mean (SD)	81^e^ (10)	87 (8)	87 (11)	91 (12)	86 (11)
Resting heart rate (beats/min), mean (SD)	67.5 (13.6)	73.7 (10.1)	75.2^d^ (11.7)	73.1 (9.8)	72.4 (12)

^a^Significantly different to Factory North (at *P* < .01).

^b^Significantly different to both office sites (at *P* < .01).

^c^Significantly different to both factory sites (at *P* < .01).

^d^Significantly different to Office North (at *P* < .01).

^e^Significantly different to all other sites (at *P* < .01).

**Figure 1:**
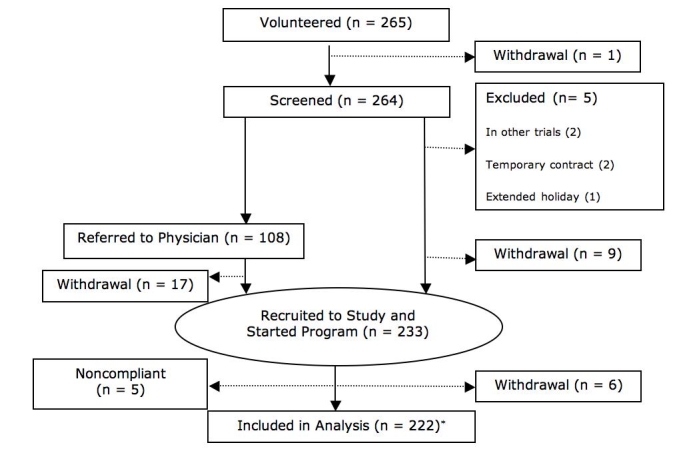
Flow chart of enrollment, withdrawal, and follow-up (*For LOCF analysis of weight change, the 5 subjects withdrawing after the program start were also included; noncompliant participants were not included in the weight LOCF analysis as no follow-up weight was recorded)

Of the 265 participants who agreed to take part, 233 started the program (Figure 1); 32 participants were excluded or withdrew in the 3-month period between initial recruitment and program start for the following reasons: did not attend screening/training session (n = 5), ineligible employment status (not permanent or leaving employment, n = 8), illness (n = 1), extended holiday (n = 1), or no physician approval received (n = 17).

The office sites had a higher total number of employees compared to the factory sites, hence the larger number of participants from these office sites. There were significant differences between the sites at baseline for age, BMI, BP, and heart rate (*P*values for testing equivalence of the sites: age, *P* = .009; BMI, *P* < .001; systolic BP, *P* = .004; diastolic BP, *P* = .001; pulse, *P* < .001), with employees from the factory sites tending to be older and having higher baseline BMI and BP (see Table 1). BP medication was recorded at baseline and at the end of the 12-week study. The use of hypertensive medication was more prevalent in employees at the Factory North site at baseline (6/43, 14%) compared to the other sites combined (5/221, 2%); 3/264 (1%) screened participants started BP medication following the baseline screening.

We compared the baseline health profiles of the 264 employees participating in the MiLife Web program to HRA data collected previously from a larger group of employees at the same sites (n = 992 total). Employees who participated in the Web program were of a similar age as those attending the HRA but had a higher mean baseline BMI (HRA: 25.0 kg/m^2^[SD 4.1]; MiLife: 27.1 kg/m^2^[SD 4.8]; *P* < .001) and higher mean baseline diastolic BP (HRA: 78.9 mmHg [SD 10.0]; MiLife: 86.0 mmHg [SD 11.0]; *P* < .001). The difference in BMI between the two populations was most noticeable at the factory sites: the mean BMI for Factory North HRA was 26.6 kg/m^2^(SD 4.2, n = 174) compared to 29.7 kg/m^2^for Factory North MiLife (SD 5.6; *P* < .001), and the mean BMI for Factory South HRA was 26.1 kg/m^2^(SD 4.2, n = 122), compared to 28.4 kg/m^2^for Factory South MiLife (SD 4.7; *P* < .001).

### Website Use

Of the 233 participants starting the program, 6 withdrew and 5 were noncompliant (no data upload or log-in throughout the 12-week period). In the remaining 222 subjects, website use remained high, with 78% (173/222) of the participants still using the website at the end of the 12-week study and 69% (153/222) continuing to use the website after the 12 weeks (Table 2).

**Table 2:** Employee website use during and following the 12-week study, by work site

	Office North	Factory North	Office South	Factory South	All Sites
Starting program, no.	67	37	79	50	233
Withdrawal during 12 weeks, no.	1	0	3	2	6
Noncompliance, no.	0	1	1	3	5
Website users at week 1, no.	66	36	75	45	222
Website users at week 12, no.	50	19	72	32	173
Website users at week 12, %	76	53	96	71	78
Website use following the 12 weeks, no.	44	14	67	28	153
Website use following the 12 weeks, %	67	39	89	62	69

**Figure 2:**
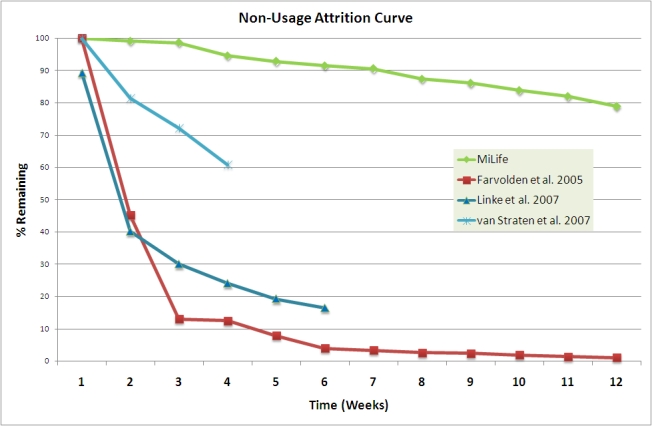
Nonusage attrition curves [27] for MiLife and Farvolden et al [31], Linke et al [32], and van Straten et al [33] eHealth interventions

Nonusage attrition data (the proportion of participants who stopped using the program and the proportion who remain) are presented in Figure 2 in comparison to reported nonusage attrition data from other Internet eHealth interventions [31-33]. Nonusage attrition rates were significantly different between the sites, with the highest use at Office South (72/75 using the Web program at 12 weeks) and the lowest use at Factory North (19/36 using the Web program at 12 weeks; *P*< .001). There was no difference in nonusage attrition rates between Factory South and Office North. Logistic regression including the baseline independent variables with site as a covariate showed that nonusage attrition was lower in both Factory North and Factory South as age increased (OR 1.07, *P*= .03). To illustrate this, 30/58 (52%) of participants 48 years old and under were using the program at 12 weeks, compared to 21/23 (91%) of participants over 48 years old. Inclusion of the dependent outcome variables in this model showed that age was no longer significant but that nonusage attrition was lower in those subjects with a greater weight change over the 12-week period, independent of site (OR 1.38, *P*= .03).

**Figure 3:**
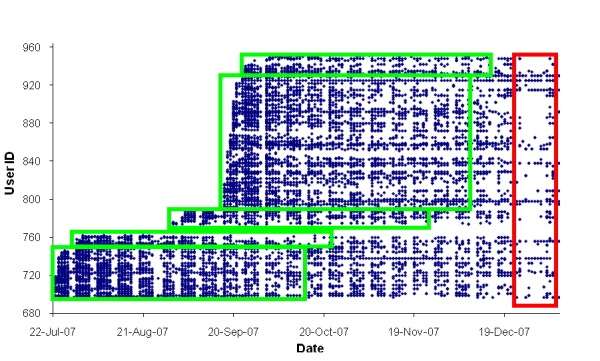
Participant’s log-in data throughout and following the study period (Each dot represents a user’s visit and log-in to the website. Green boxes are the website visits recorded during the 12-week study period. Data within the red box were recorded over the 2-week Christmas holiday period when the work sites were closed.)

Log-in data are shown in Figure 3). Spaces between weeks can be clearly seen, indicating that most participants were using the website on weekdays and not weekends, with 7381 (92%) of the 8067 log-ins recorded during the 12-week study occurring between Monday and Friday. Data recorded over the Christmas holiday period also suggest that most users did not use the program on non-workdays, although 69 (31%) of the 222 website users did log-in to the program at least once during this 2-week break. Continued use of the program outside of the study period can also be seen on this graph.

**Figure 4:**
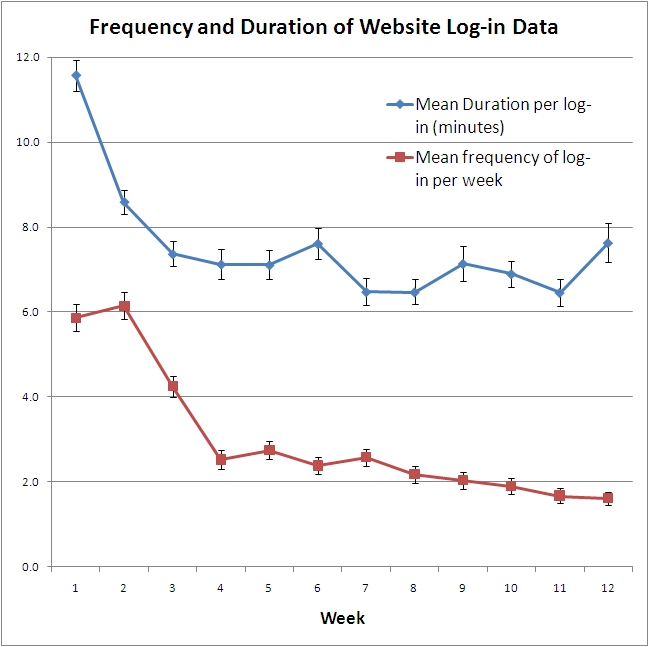
Mean website log-in duration and frequency throughout the study period (Data points are means with standard error and are presented for all employees as there was no significant difference between the sites based on ANOVA.)

**Figure 5:**
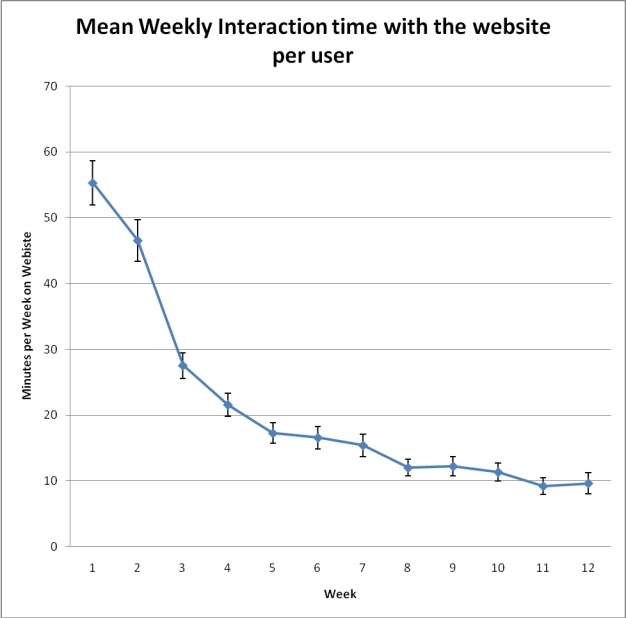
Mean interaction time with the website throughout the study period (Data points are means with standard error and are presented for all employees as there was no significant difference between the sites based on ANOVA.)

During the first 2 weeks of the study, participants were spending more time on the website per log-in compared to the subsequent weeks (mean week 1: 11.6 minutes; mean week 2: 8.6 minutes; Figure 4). After this initial period, the mean time per website log-in (weeks 3-12) was approximately 7 minutes. The total website interaction time per week was also collected for each user. As both the frequency of use and the time per visit dropped with ongoing program use, the total interaction time with the website (Figure 5) was higher in the first 4 weeks, dropping to a mean value between 10 and 20 minutes per week for the remainder of the study. Multivariate linear regression showed that the log total interaction time over the 12 weeks was higher in participants with the greatest weight loss (slope = .082; *P*< .001). To illustrate the magnitude of this slope (mean log duration 2.472), each additional kilogram of weight loss was approximately equivalent to an extra hour of program use over the 12-week period. Gender was also associated with total interaction time over the 12 weeks, with women spending, on average, 200 more minutes interacting with the Web program over the 12-week period (*P*= .002).

### Weight Data

**Figure 6:**
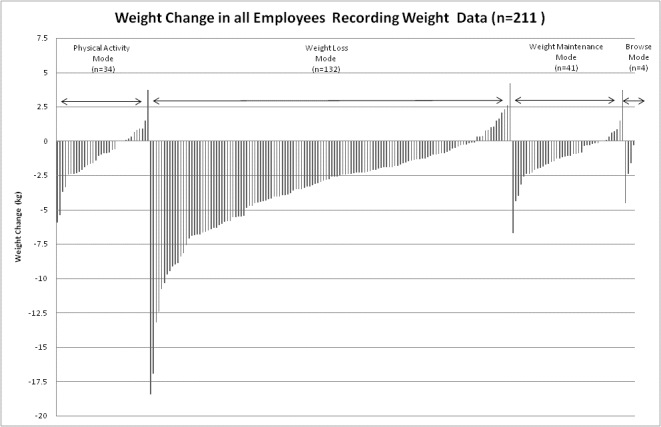
Weight change from baseline using the LOCF and the mode in which the most time was spent for each employee uploading weight data during the 12-week program

Of the 228 employees using the program (222 starting the program plus 6 withdrawals during the 12-week study), 211 (93%) uploaded weight data that could be used to determine weight change during the study period using the LOCF. The mean weight change in this group was −2.6 kg (SD 3.2; *P*< .001; Figure 6).

**Figure 7:**
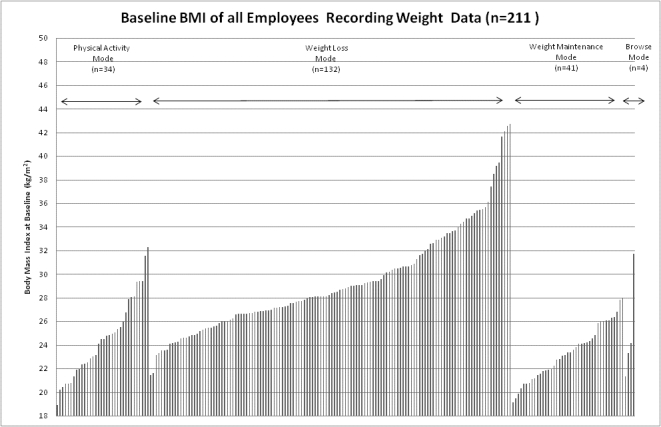
Baseline BMI and mode in which the most time was spent for each employee uploading weight data during the 12-week program

Mean weight loss was higher in those employees who spent most of the 12-week period in the weight loss mode (132/212, 63%; mean weight change −3.5 kg, SD 3.6). No statistical comparison was conducted between modes since subjects could switch modes during the study period. There was no significant difference in weight change between the sites, but there was a significant inverse association between baseline BMI and the amount of weight lost over the 12-week period (−0.284, *P* < .001), indicating that employees with a higher starting BMI, on average, had greater weight loss during the study period. Figure 7 shows the baseline BMI distribution in each of the modes, suggesting that subjects with a higher baseline BMI spent most of their time during the 12-week study in the weight loss mode.

### Physical Activity (PA) Data

The accelerometer-recorded levels of PA were highly variable between individuals in the group, with values ranging from 12 to 714 minutes of moderate or above PA per person per week. The average recorded level for the group was 173 minutes (SE 12.8) of moderate or above PA per week.

### Choice of Goals

At all sites, weight loss was the most popular mode. Of the 228 website users (including those who withdrew during the 12-week study), 138 (61%) spent the most time in weight loss mode, 46 (20%) spent the most time in weight maintenance mode, 39 (17%) spent the most time in the PA only mode, and 4 (2%) spent the most time in the nonactive browse mode of monitoring without goal and target setting.

**Figure 8:**
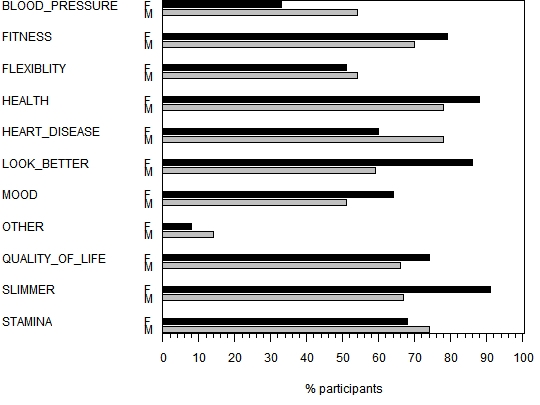
Frequency of choice of the listed goals prior to beginning the 12-week Web program (F = women, M = men)

At the start of the program, each participant was asked to select one or more goals that he or she would most want from a list on the website. Research has shown that people with a strongly desirable goal are more likely to enact their intentions to perform a health behavior [34]. The list consisted of the following goals: improve fitness, increase flexibility, improve health, reduce risk of heart disease, reduce blood pressure, look better, improve mood, improve quality of life, feel slimmer, improve stamina, other. Figure 8 shows the frequency of selection of these items by gender. The most frequently chosen goals for men were “health” and “heart disease,” while the most frequently chosen goal for women was “feel slimmer.” Men were more likely than women to select “heart disease” and “blood pressure” as reasons for participating, while women were more likely to select “look better” and “feel slimmer.”

### Blood Pressure Data

At the 12-week BP assessment, some employees at each site were lost to follow-up (Office South, 28%; Office North, 35%; Factory South, 35%; Factory North, 51%). Logistic regression with site, age, and gender as covariates showed that participants with a higher baseline BP were more likely to attend the follow-up (P= .047).

The high level of participants lost to follow-up was possibly influenced by the proximity of these measures to the Christmas holiday period. As a result, data have been aggregated for all employees who returned for a 12-week BP measure (n = 135, excluding those on hypertensive medication). The mean baseline BP in this group was 129/86 mmHg (SD 15/10, range 94/64 to 181/119), and the mean 12-week BP was 128/80 mmHg (SD 15/10, range 95/59 to 164/100). There was a significant reduction in diastolic BP (−5.9 mmHg, SD 9.9;P< .001).

### Sleep Data

Data from the Pittsburgh Sleep Quality Index (PSQI) questionnaire collected at baseline and 12 weeks (n = 93 completed) suggested an increase in sleep quality overall, corresponding to a decrease in the global PSQI score (*P* = .004). This was particularly evident in the following PSQI components: self-assessed overall sleep quality (*P* < .001), hours of actual sleep achieved (*P* = .01), ease of both maintaining attention and/or enthusiasm for everyday tasks (*P* = .006).

### Program Evaluations

Data collected from the exit questionnaires (n = 130) showed that 101 employees (78%) found the website very easy to use, with the most useful tools listed as those providing PA analysis, planning, and information. Many employees liked wearing the PA monitor and found that having it on served as a constant reminder to keep to the program. The site was seen as informative, motivating, and helpful. The PA and weight charts were thought particularly helpful as they enabled participants to monitor their progress and played an important role in providing feedback and motivation. The low response rate (130/222 website users, 59%) to the exit questionnaire may have been influenced by the proximity to the holiday period.

The OH staff at the work sites who responded to the survey (n = 6) agreed that “the study had been a supportive program in the company objective”of vitalizing employee health. Several of the OH staff commented that the study had been a positive initiative that participants had found enjoyable and rewarding and which should be encouraged. Employee participation in the program resulted in some extra work for OH staff in answering participants queries, although OH staff were generally happy with the resources they had received from the study team to support participants.

Feedback from managers at the work sites (n = 6) was overall very positive, and managers received positive feedback from participants. Managers noted that the study appeared to have been beneficial in the workplace, and participation may even have led to increased job satisfaction in some instances. Managers were also in agreement that they would encourage future staff participation in the Web program and would recommend participation in the program to other sites.

## Discussion

### Principal Results

The study was designed to test the level of Web program engagement over a 12-week period for a wide range of employees recruited at four work sites in the United Kingdom. Work sites were chosen that were geographically and demographically different in order to evaluate if engagement and outcomes varied by location, baseline demographic and health measures, or level of interaction with computers during work hours.

This study showed that a Web-based PA and weight management program designed to include components known to be effective (a structured approach to modifying energy balance, the use of cognitive-behavioral strategies such as self-monitoring, and individualized feedback and support [3]) was appealing to employees at both office and manufacturing sites. The combination of the automated Web-based coaching program with monitoring devices to record PA level and weight data produced high levels of engagement with the program both during and following the initial 3-month period. Health improvements were also observed, as indicated by changes in body weight, BMI, blood pressure, and sleep. The program appealed equally to both men and women, and shift work was not a barrier to participation.

A key difference in this study compared with our previous studies [4,5] was that participants received no payment for taking part in this study. The 12% employee participation rate is similar to the 10% employee participation rate reported in another Web-based workplace program [35]; however, in that study, employees were offered financial incentives to take part.

Also, rather than an RCT, the current study was conducted in a more naturalistic setting in which a branded commercial program (MiLife) was offered to employees as a benefit in collaboration with their company’s OH professionals. One limitation of this approach was the lack of a control group. Subsequent use of the program in an employee wellness setting may provide the opportunity to test engagement with the program against alternative weight management and PA initiatives available to those employees. This would build on the insights generated in this research by using an RCT efficacy design and allowing a full intent-to-treat and per protocol statistical analysis.

### Participant Characteristics

Employees who participated in the Web program had a higher average diastolic BP and BMI compared to employees previously taking part in an HRA at the same site. This was most noticeable at the factory sites. This does not mean that the Web program enrolled all high-risk individuals, as not all employees take part in HRAs [36], but it does indicate that the program attracted those employees who would benefit most from PA and weight management. This can also be seen from the number of employees (41%) failing the PAR-Q or the number with hypertension who needed physician approval prior to starting the Web program. The number of withdrawals in this physician-referred group (17/108) was higher than that in the non-referred group (9/151). However, we did not collect data on whether it was the referral process itself that was responsible for this. Future studies that collect more information on physician referrals and uptake of employee programs following the referral would be very useful in determining the effect of referrals on wellness programs.

Further comparison of the baseline demographic data in this adult employee population with the profiles of participants using a Dutch Web-based health promotion program available to the Dutch general public at no cost [37] suggests that the MiLife program recruited a higher proportion of males and participants with a higher average BMI (27.1 kg/m^2^). Both programs were Web-based PA and weight management programs, although it is unclear if the difference in the user profiles was due to differences in the programs or to the populations targeted by the interventions. The Dutch Web-based program did conclude that obese people were more likely to participate in Web-based programs, possibly because of the non-stigmatizing way of addressing body weight through the Internet.

### Engagement

Nonusage attrition rates were much lower with MiLife than with other Web-based eHealth interventions that have reported this data [31-33]. This may be due to the combination of monitoring devices with the Web program. The inclusion of weight management may also be a driver for continued participation [37]. A weight management Web-based program reporting similar levels of engagement is one described by Stevens et al [38] for weight loss maintenance, which required users to log-in once a month. While the MiLife program has more interaction time with the participant through the data upload and self-monitoring process, both programs share the common feature of automated email reminders to the participants to log-in. Attrition was higher in the Factory North site, and it is possible that the weekly email reminders were less effective here, although attrition in the Factory South site was not different than at the office sites. Use of personalized mail may further enhance engagement with Web-based eHealth interventions [39], especially where PC access may be limited during working hours. The higher levels of nonusage attrition observed in Factory North may also have influenced the higher loss to follow-up for the 12-week BP measure at this site [27]. This reinforces the value of early intervention, if, for example, the number of log-ins or the frequency of data upload declines, to reduce attrition rates.

Recent reviews including more than 50 studies of Internet-based programs for PA and dietary behavior change [40,41] showed that only 6 programs incorporated an objective PA monitoring device. Three of these studies used accelerometers [42-44], and 3 used pedometers [45-47].

Norman et al [41] highlight in their review that an issue for eHealth interventions is getting participants to use the interactive technology often enough to receive an optimal dose of the intervention and that where utilization and dose are higher, better behavior change is observed. They also discuss how, by assessing smaller milestones more frequently, the technology can automatically create slightly more challenging goals to enhance the likelihood of achieving longer term behavior change goals. We did observe in this study that data capture from the monitoring devices (weight and PA) was significantly greater than data capture from the user, who was required to input data manually into the website and self-report (eg, calorie consumption).

Reinforcement and positive feedback are also an important part of the program, and the PA and weight graphs and charts were the most liked part of the program by users. Where positive feedback is not received for effort, users may be less likely to engage with the program. In a pedometer-based walking program, those users who did not receive positive feedback for all of their effort were 5 times more likely to fail to wear the pedometer compared with a group for whom total effort was recognized [48].

Therefore, in this program, the use of simple monitoring devices to continuously record PA data and weight data combined with automated data upload to the website and positive feedback may have had a number of effects. It is likely that this approach influenced the high levels of data capture, the utilization and engagement rates observed, and also the likelihood of achieving the behavior change. The combination of monitoring devices with a Web-based program is not without challenges, and we did experience some initial hardware reliability issues. This was anticipated to an extent, and part of the study design was to test the robustness of the hardware in such a large group of subjects. While replacement devices were issued to any employee experiencing technical problems, feedback from the exit questionnaires indicated that hardware problems did influence the user experience for some participants. However, Web program use remained high in these employees.

Analysis of the mean interaction time with the website suggests that there was a learning period in the first few weeks, with users spending more time on the website, finding the tools, and navigating the site. Typically by the fourth week of the program, subjects were interacting with the website for 10-20 minutes per week, and the total time spent on the website over the 12 weeks was associated with the amount of weight lost.

The time spent on the website per week is similar to our previously reported work [5], in which subjects were found to interact with the program for an average of 10-12 minutes per week compared to an average of 55 minutes in the first week. The log-in data clearly show that many employees were using the website during the week but less so during weekends, possibly indicating that much of the website interaction time was during their working day. Data from the WebSense 2006 Web@Work Survey [49] suggest that 61% of employees who utilize a work-owned Internet connection spend, on average, 3 hours per week surfing non-work-related websites during the workday. While the effect of this on the company is unclear, there is a clear benefit to the individual and to the company of 10-20 minutes per week of Internet use for improved employee health. Positive effects on weight, PA level, BP, and sleep were observed in this study. Further studies are planned to investigate these outcomes in a randomized controlled study and over a longer period of time.

### Conclusions

This study suggests that the MiLife Web-based program designed to support PA and weight management and utilizing simple monitoring devices for weight and PA can be successfully deployed in both office and manufacturing sites. The program received positive feedback from both OH staff and managers at each site. Most importantly, the program appealed to and engaged those employees who would most benefit from changes in PA and weight management, with many employees enjoying the experience, improving their health parameters, and returning to follow a second 3-month program with no financial incentive.

## Multimedia Appendix

Image of the interactive website and of the monitoring devices

## Abbreviations

BMI: body mass index
BP: blood pressure
HRA: health risk appraisal
LOCF: last observation carried forward
MET: metabolic equivalent
OH: occupational health
PA: physical activity
PAR-Q: Physical Activity Readiness Questionnaire
PC: personal computer
PSQI: Pittsburgh Sleep Quality Index
RCT: randomized controlled trial
