# Supplementary figures and images for: Rates and Determinants of Uptake and Use of an Internet Physical Activity and Weight Management Program in Office and Manufacturing Work Sites in England: Cohort Study
Source: J Med Internet Res. 2008 Dec 31;10(4):e56. doi: 10.2196/jmir.1108 (PMC2629365; doi:10.2196/jmir.1108)

## Slide 1
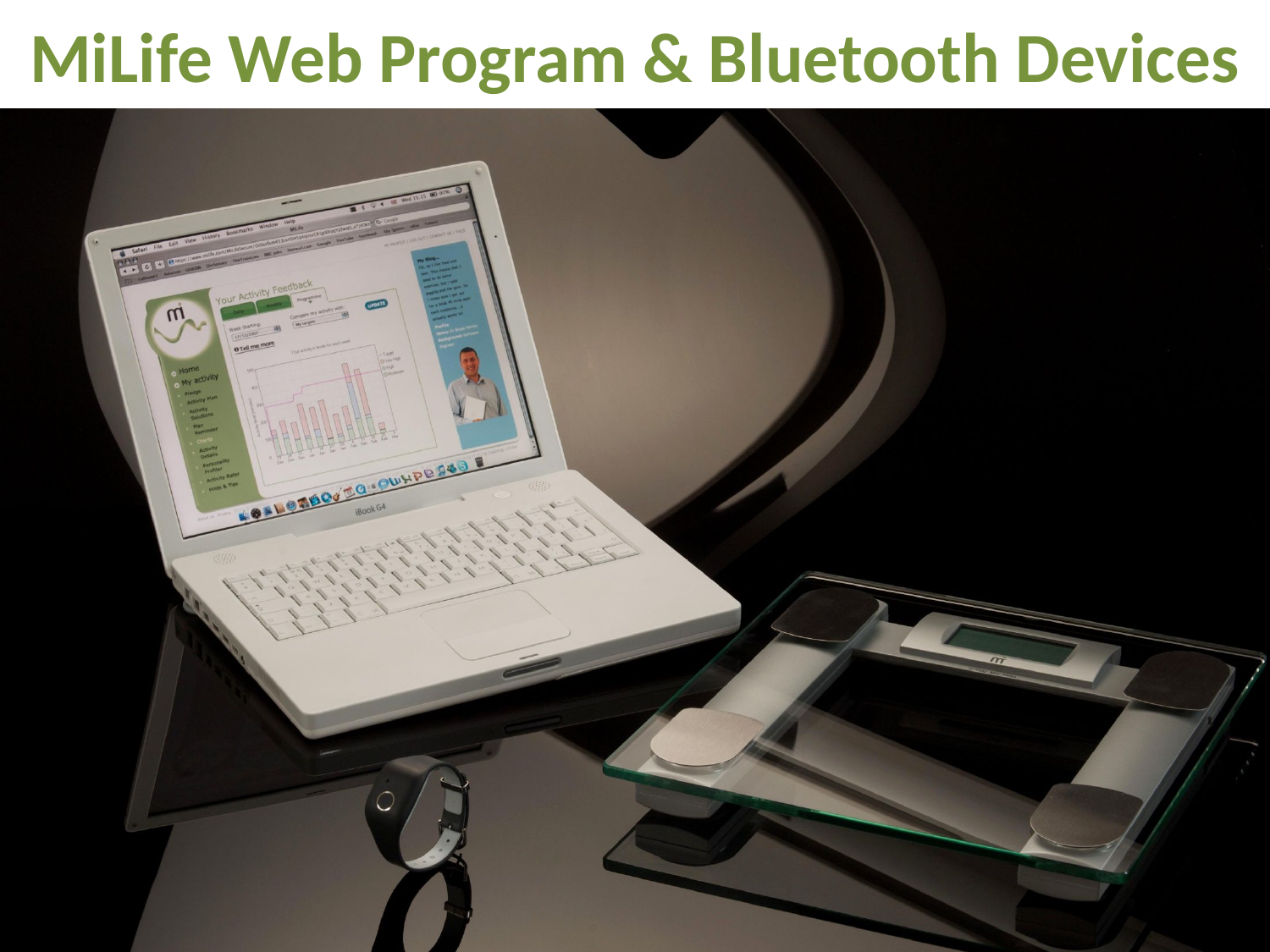

# MiLife Web Program & Bluetooth Devices

Supplement: Supplementary file 1 [file jmir_v10i4e56_app.ppt]
